# Supplementary material for: Shapley variable importance cloud for interpretable machine learning
Source: Patterns (N Y). 2022 Feb 22;3(4):100452. doi: 10.1016/j.patter.2022.100452 (PMC9023900; doi:10.1016/j.patter.2022.100452)
Supplement: Document S1. Figures S1–S3 [file mmc1.pdf]

Patterns, Volume *3*

Supplemental information

Shapley variable importance cloud

for interpretable machine learning

Yilin Ning, Marcus Eng Hock Ong, Bibhas Chakraborty, Benjamin Alan Goldstein, Daniel Shu Wei Ting, Roger Vaughan, and Nan Liu

Figure S1. Visual summary of recidivism prediction study results from the VIC analysis. VIC values were subtracted 1 such that zero value or lower indicate unimportance. (A) Average values from the 350 nearly optimal models. Variable ranking was similar to that based on average ShapleyVIC values. (B) Distribution of VIC values (subtracted 1) and the corresponding model performance.

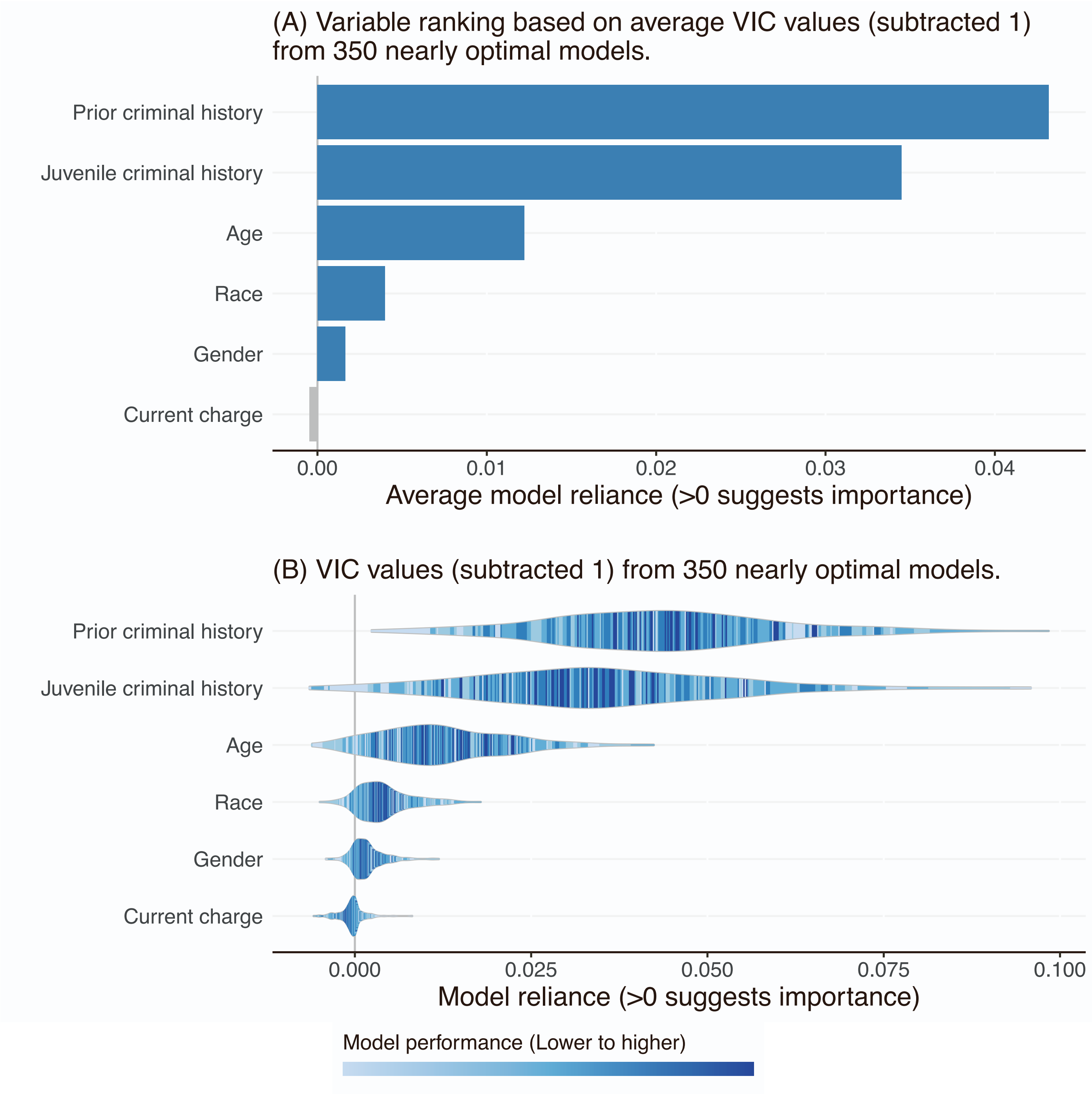

Figure S2. Spearman correlation among variables in the MIMIC study, arranged by variance inflation factor from the minimum-loss model. Absolute values larger than 0.4 are colored (in blue for positive correlation and red for negative correlation).

	Glucose	WBC	SpO2	Platelet	Temperature	Respiration rate	Age	Lactate	Potassium	Heart rate	BUN	Creatinine	SBP	DBP	Anion gap	MAP	Sodium	Bicarbonate	Chloride	Hematocrit
WBC	0.14																			
SpO2	0.01	0.03																		
Platelet	0.04	0.29	−0.05																	
Temperature	0.01	0.12	0.06	0																
Respiration rate	0.05	0.11	−0.25	0.09	0.12															
Age	0.07	0.01	−0.06	−0.05	−0.16	0.07														
Lactate	0.12	0.12	0.08	−0.11	0.02	0.01	−0.02													
Potassium	0.08	0.08	0.01	−0.03	−0.07	−0.05	0.08	0.05												
Heart rate	0.08	0.17	−0.05	0.03	0.27	0.29	−0.21	0.1	0											
BUN	0.14	0.03	−0.07	−0.08	−0.18	0.11	0.34	0.03	0.3	−0.07										
Creatinine	0.1	0.01	−0.06	−0.08	−0.11	0.08	0.17	0.07	0.29	−0.08	0.74									
SBP	0.08	−0.06	0.01	0.07	0.05	−0.03	0	−0.04	−0.08	−0.09	−0.03	0								
DBP	0.01	−0.07	−0.03	0.07	0.02	0.01	−0.36	0	−0.11	0.2	−0.2	−0.11	0.49							
Anion gap	0.16	0.11	−0.07	0.14	−0.04	0.16	−0.02	0.18	0.1	0.09	0.34	0.42	0.07	0.07						
MAP	0.06	−0.04	0.03	0.06	0.05	−0.03	−0.22	0.01	−0.11	0.09	−0.16	−0.1	0.76	0.85	0.07					
Sodium	−0.06	−0.04	0.01	0	0.03	0.02	0	0.01	−0.19	−0.05	0.01	0	0.11	0.08	0	0.1				
Bicarbonate	−0.06	−0.13	−0.12	0.09	−0.01	−0.07	0.04	−0.17	−0.11	−0.13	−0.13	−0.18	0.11	0.05	−0.42	0.06	0.11			
Chloride	−0.08	0.03	0.19	−0.19	0.06	−0.07	0	0.05	−0.01	0.01	−0.09	−0.1	−0.07	−0.06	−0.24	−0.04	0.54	−0.41		
Hematocrit	0.03	0.1	−0.15	0.13	0.01	0	−0.15	0.06	−0.04	−0.06	−0.2	−0.08	0.12	0.28	0.14	0.23	0.12	0.1	−0.11	
Hemoglobin	0.02	0.08	−0.13	0.09	0.03	−0.02	−0.17	0.06	−0.08	−0.07	−0.23	−0.11	0.13	0.29	0.12	0.25	0.1	0.08	−0.1	0.97

BUN: blood urea nitrogen; DBP: diastolic blood pressure; MAP: mean arterial pressure; SBP: systolic blood pressure; SpO_2: peripheral capillary oxygen saturation; WBC: white blood cell.

Figure S3. Visual summary of MIMIC study results from the VIC analysis. VIC values were subtracted 1 such that zero value or lower indicate unimportance. (A) Average values from the 350 nearly optimal models. Five of the top seven variables (i.e., except MAP and chloride) by average VIC values were also ranked top seven by average ShapleyVIC values. (B) Distribution of VIC values (subtracted 1) and the corresponding model performance.

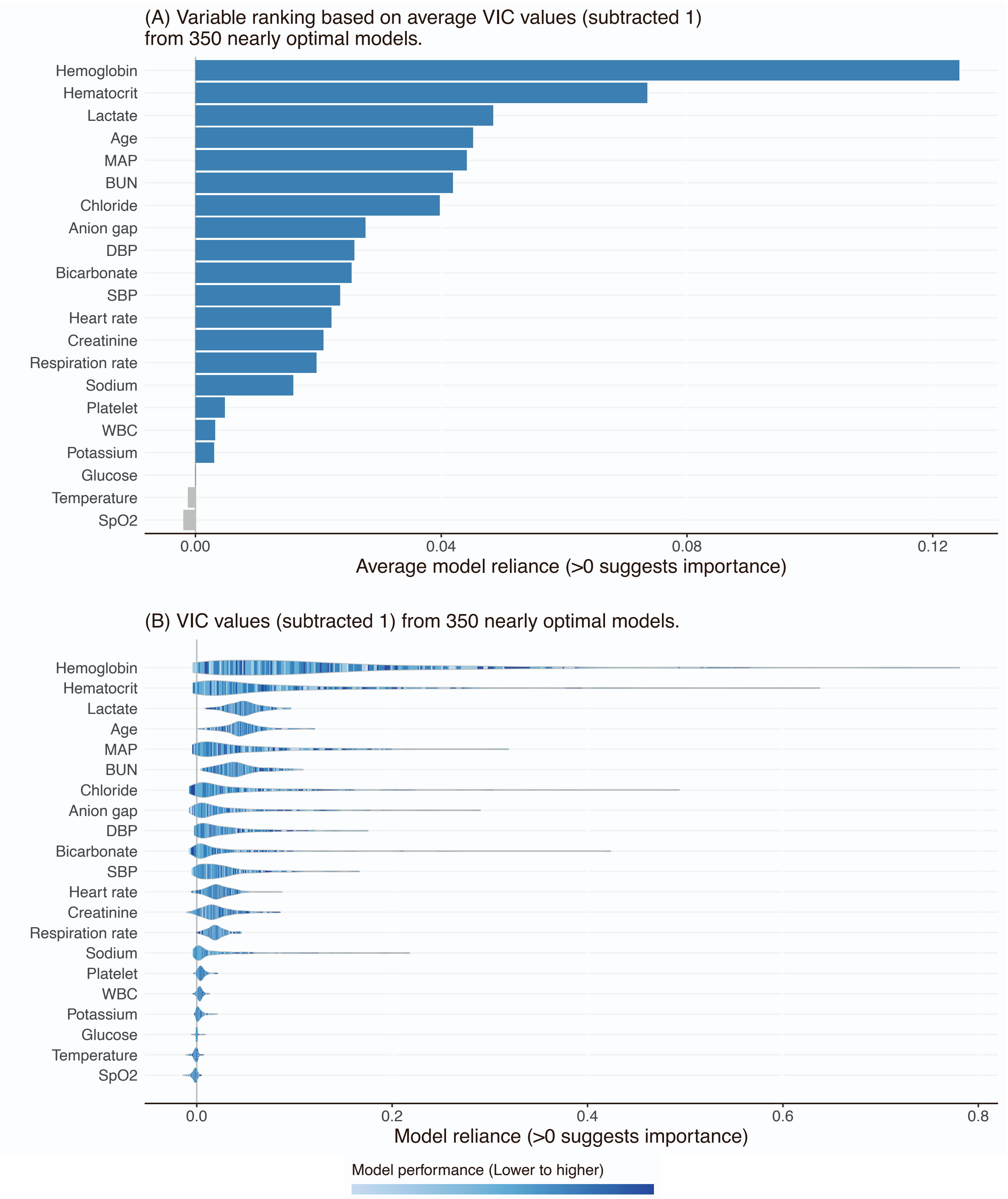

BUN: blood urea nitrogen; DBP: diastolic blood pressure; MAP: mean arterial pressure; SBP: systolic blood pressure; SpO_2: peripheral capillary oxygen saturation; WBC: white blood cell.
