# Supplementary material for: Shapley variable importance cloud for interpretable machine learning
Source: Patterns (N Y). 2022 Feb 22;3(4):100452. doi: 10.1016/j.patter.2022.100452 (PMC9023900; doi:10.1016/j.patter.2022.100452)
Supplement: Document S2. Article plus supplemental information [file mmc2.pdf]

Patterns

Article

Shapley variable importance cloud for interpretable machine learning

Graphical abstract

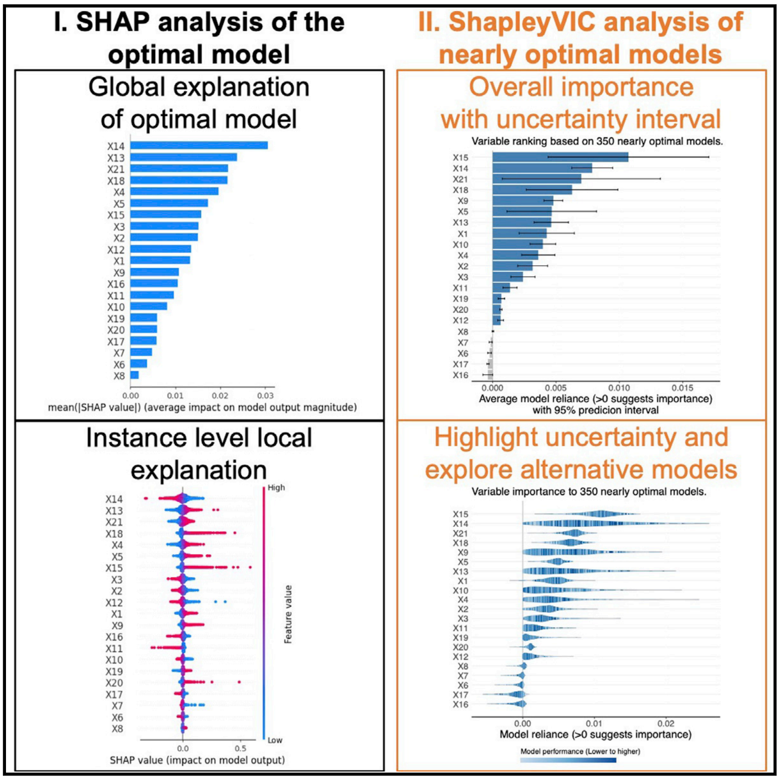

Authors

Yilin Ning, Marcus Eng Hock Ong, Bibhas Chakraborty, Benjamin Alan Goldstein, Daniel Shu Wei Ting, Roger Vaughan, Nan Liu

Correspondence

liu.nan@duke-nus.edu.sg

In brief

Variable importance assessment is important for interpreting machine learning models. Current practice in interpretable machine learning applications focuses on explaining the final models that optimize predictive performance. However, this does not fully address practical needs, where researchers are willing to consider models that are "good enough" but are easier to understand or implement. Our work fills this gap by extending the current method to a set of "good models" for comprehensive and robust assessments and demonstrates the benefits in multiple domains.

Highlights

- Comprehensive global variable importance assessments beyond final (optimal) models
- Integrates with SHAP to complement current interpretable machine learning research
- ShapleyVIC quantifies uncertainty in variable importance for rigorous assessments
- ShapleyVIC visualizes uncertainty to explore good models with specific properties

Ning et al., 2022, Patterns 3, 100452
April 8, 2022 © 2022 The Author(s).
https://doi.org/10.1016/j.patter.2022.100452

CellPress

Patterns

CellPress
OPEN ACCESS

Article

Shapley variable importance cloud for interpretable machine learning

Yilin Ning,[1] Marcus Eng Hock Ong,[2,3,4] Bibhas Chakraborty,[1,2,5,6] Benjamin Alan Goldstein,[2,6] Daniel Shu Wei Ting,[1,7,8] Roger Vaughan,[1,2] and Nan Liu[1,2,3,8,9,10,*]

[1]Centre for Quantitative Medicine, Duke-NUS Medical School, 8 College Road, Singapore 169857, Singapore
[2]Programme in Health Services and Systems Research, Duke-NUS Medical School, 8 College Road, Singapore 169857, Singapore
[3]Health Services Research Centre, Singapore Health Services, 20 College Road, Singapore 169856, Singapore
[4]Department of Emergency Medicine, Singapore General Hospital, 1 Hospital Crescent Outram Road, Singapore 169608, Singapore
[5]Department of Statistics and Data Science, National University of Singapore, 6 Science Drive 2, Singapore 117546, Singapore
[6]Department of Biostatistics and Bioinformatics, Duke University, 2424 Erwin Road, Durham, NC 27710, USA
[7]Singapore Eye Research Institute, Singapore National Eye Centre, 11 Third Hospital Avenue, Singapore 168751, Singapore
[8]SingHealth AI Health Program, Singapore Health Services, 10 Hospital Boulevard, Singapore 168582, Singapore
[9]Institute of Data Science, National University of Singapore, 3 Research Link, Singapore 117602, Singapore
[10]Lead contact
*Correspondence: liu.nan@duke-nus.edu.sg
https://doi.org/10.1016/j.patter.2022.100452

THE BIGGER PICTURE With the wide use of machine learning models in decision making, various explanation methods have been developed to help researchers understand how each variable contributes to predictions. However, the current explanation approach focuses on explaining the final (often best performing) models, ignoring the fact that in practice, researchers are willing to consider models that are "good enough" and are easier to understand and/or implement. We propose the Shapley variable importance cloud to address this practical need by extending the current explanation approach to a set of "good models," which pools information across models to derive a more reliable measure for overall variable importance. Moreover, we analyze and visualize the uncertainty of variable importance across models, which enables rigorous statistical assessments and helps discover alternative models with preferrable properties.

1 2 **3** 4 5 **Development/Pre-production:** Data science output has been rolled out/validated across multiple domains/problems

SUMMARY

Interpretable machine learning has been focusing on explaining final models that optimize performance. The state-of-the-art Shapley additive explanations (SHAP) locally explains the variable impact on individual predictions and has recently been extended to provide global assessments across the dataset. Our work further extends "global" assessments to a set of models that are "good enough" and are practically as relevant as the final model to a prediction task. The resulting Shapley variable importance cloud consists of Shapley-based importance measures from each good model and pools information across models to provide an overall importance measure, with uncertainty explicitly quantified to support formal statistical inference. We developed visualizations to highlight the uncertainty and to illustrate its implications to practical inference. Building on a common theoretical basis, our method seamlessly complements the widely adopted SHAP assessments of a single final model to avoid biased inference, which we demonstrate in two experiments using recidivism prediction data and clinical data.

INTRODUCTION

Machine learning (ML) methods has been widely used to aid high-stakes decision making, e.g., in healthcare settings.[1,2] While ML models achieve good performance by capturing data patterns through complex mathematical structures, such complexity results in "black box" models that hide the underlying mechanism. The inability to assess the connection between

This is an open access article under the CC BY license (http://creativecommons.org/licenses/by/4.0/).

variables and predictions makes it difficult to detect potential flaws and biases in the resulting prediction models and limits their uptake in real-life decision making.[3–7] The growing research on interpretable ML (IML), also interchangeably referred to as explainable artificial intelligence in the literature, improves the usability of ML models by revealing the contribution of variables to predictions.[6–10]

A lot of effort in IML has been put into "post hoc" explanations that quantify the variable impact on a model while leaving the model a black box.[7,9,10] For example, the random forest[11] was developed with a permutation importance that evaluates reductions in model performance after removing each variable, which partially contributes to its wide adoption in practice.[10] A recent study introduced a similar permutation-based model-agnostic approach, termed model reliance, that provides global explanations for any ML models.[12] Current IML applications are dominated by two local model-agnostic explanation approaches:[13] the local interpretable model-agnostic explanations (LIME)[14] explains individual predictions by locally approximating them with interpretable models, and the Shapley additive explanations (SHAP)[15] attributes a prediction among variables by considering it as a cooperative game. These two methods are connected: both linear LIME and SHAP are additive feature attribution methods, where SHAP provides a more disciplined approach for setting the weighting kernels involved, resulting in desirable properties that are not guaranteed by the heuristic approach used in LIME.[15]

A desirable property of SHAP is that in addition to locally explaining individual predictions, the mean absolute SHAP values can provide heuristic measures of variable importance to overall model performance,[15,16] and a formal global extension, i.e., Shapley additive global importance (SAGE),[16] was developed recently. However, by leaving the black box unopened, these methods do not fully reveal the mechanism of the models, e.g., why do some variables contribute more to the predictions than others?[7] Ante hoc IML methods address this by developing inherently interpretable models, e.g., recent works[17–19] proposed ML approaches to build sparse scoring systems based on simple regression models that had good discriminative ability. By integrating considerations such as variable importance into model-building steps, these methods support direct inference on the importance of variables to the outcome.

While most IML approaches focus on optimal (e.g., loss minimizing) models, a recent work[20] broadened the scope to include a wider range of models that are "good enough." These nearly optimal models are highly relevant to practical questions, e.g., can an accurate yet expensive biomarker be replaced with other variables without strongly impairing prediction accuracy?[20] To systematically address such questions, Dong and Rudin[20] proposed a variable importance cloud (VIC) that provides a comprehensive overview of variable contributions by analyzing the variability of variable importance across a group of nearly optimal models and found an overclaim of the importance of race to the criminal recidivism prediction in post hoc assessments.

VIC is the first to demonstrate the benefit of extending global interpretation to include nearly optimal models, which is not available from the state-of-the-art SHAP method or the recent global extension via SAGE. However, VIC was developed from the permutation importance,[12,20] hence leaving a gap between theoretical developments and current applications based on Shapley values. We propose a Shapley variance importance cloud (ShapleyVIC) that extends SHAP to higher-level global interpretations by integrating the latest development in Shapley-based variable importance measures with the recently proposed VIC framework. ShapleyVIC contributes to IML research by providing additional insights into variable importance than post hoc SHAP assessments, which easily integrates with SHAP to provide a comprehensive model explanation on the local level for individual instances, on the global level for the optimal model, and finally across nearly optimal models for overall assessments. In addition, ShapleyVIC explicitly quantifies the variability of variable importance across models to enable formal inference and conveys it through novel visualizations. We demonstrate the use of ShapleyVIC and its practical implications as a complement to SHAP analysis in two experiments, where experiment 1 revisits the previous analysis of criminal recidivism prediction[20] and experiment 2 assesses variable contributions when predicting mortality using real-life clinical data.

RESULTS

Analytical results

The VIC framework has two key components: a global importance measure to quantify the reliance of a model on each variable, and a formal definition of nearly optimal models. In VIC, the former was quantified using a permutation-based importance measure, and the latter was defined by the Rashomon set.[20] Following the VIC framework, our proposed ShapleyVIC extends the widely used Shapley-based variable importance measures beyond final models for a comprehensive assessment and has important practical implications. ShapleyVIC uses the same definition of nearly optimal models as VIC but quantifies model reliance on variables using SAGE, a Shapley value for global importance. In the following subsections, we describe the permutation importance and Shapley values, introduce the definition of nearly optimal models and the corresponding VIC, define ShapleyVIC with explicit variability measures to support inference, and describe our practical solutions to some challenges in implementation.

Global importance measures

Let Y denote the outcome and let $X_D = \{X_1, \ldots, X_d\}$ collectively denote d variables, where $D = \{1, \ldots, d\}$ is the set of all variable indices. A model of Y built using the d variables is denoted by $f(X_D)$, with expected loss $E\{L(f(X_D), Y)\}$. Fisher and team[12] proposed a permutation-based measure of variable contribution, referred to as model reliance (MR). The MR of variable X_j ($j \in D$) is the increase in expected loss when the contribution of this variable is removed by random permutation:

$$mr_j(f) = \frac{E\left\{L\left(f\left(X_{D\setminus\{j\}}, X_j'\right), Y\right)\right\}}{E\{L(f(X_D), Y)\}},$$

where $X_{D\setminus\{j\}}$ denotes the set X_D after excluding X_j, and X_j' follows the marginal distribution of X_j. $mr_j(f) = 1$ suggests model f does not rely on X_j, and larger $mr_j(f)$ indicates increased reliance.

Although straightforward and easy to implement, the permutation approach does not account for interactions among variables, as it removes one variable at a time.[16,21] Shapley-based

explanations account for this by viewing variables as players in a cooperative game[15,16] and measures the impact of variable X_j on model f based on its marginal contribution when some variables, $X_S \subset X_D$, are already present. The Shapley values are defined as:

$$\varphi_j(w) = \frac{1}{d} \sum_{S \subseteq \{D \setminus \{j\}\}} \binom{d-1}{|S|}^{-1} [w(S \cup \{j\}) - w(S)]. \quad \text{(Equation 1)}$$

$w(S)$ quantifies the contribution of subset X_S to the model, which is defined differently for different types of Shapley-based variable importance measures and will be explicitly defined below for SHAP and SAGE. $|S|$ denotes the number of variables in this subset, and $\binom{d-1}{|S|}$ is the number of ways to choose $|S|$ variables from $X_{D \setminus \{j\}}$. $\varphi_j(w) = 0$ indicates no contribution, and larger values indicate increased contribution.[16]

When $w(S)$ is the expectation of a single prediction, i.e., $w(S) = v_{f,x}(S) = E[f(X_D|X_S = x_S)]$, $\varphi_j(v_{f,x})$ gives the SHAP value for local explanation.[15] Absolute SHAP values reflect the magnitude of variable impact, and the signs indicate the direction; therefore, the mean absolute SHAP value may be used as a heuristic global importance measure.[15,16]

When $w(S)$ is the expected reduction in loss over the mean prediction by including X_S, i.e., $w(S) = v_f(S) = E\{L(E[f(X_D)], Y)\} - E\{L(f(X_D|X_S = x_S), Y)\}$, $\varphi_j(v_f)$ is the SAGE value for a formal global interpretation.[16] Our proposed ShapleyVIC follows the VIC approach to extend the global and model-agnostic SAGE across models.

Nearly optimal models and VIC

Suppose $f^*(X_D)$ is the optimal model that minimizes expected loss among all possible f from the same model class, F (e.g., the class of logistic regression models). Dong and Rudin[20] proposed extending the investigation of variable importance to a Rashomon set of models with nearly optimal performance (in terms of expected loss):

$$R(\varepsilon, f^*, F) = \{f \in F | E\{L(f(X_D), Y)\} \le (1+\varepsilon) E\{L(f^*(X_D), Y)\}\},$$

where "nearly optimal" is defined by the small positive value ε, e.g., $\varepsilon = 5\%$. Using $MR(f) = \{mr_1(f), \ldots, mr_d(f)\}$ to denote the collection of MR for model f, the VIC is the collection of MR functions of all models in the Rashomon set, $R = R(\varepsilon, f^*, F)$, defined above:[12,20]

$$VIC(R) = \{MR(f) : f(X_D) \in R(\varepsilon, f^*, F)\}.$$

VIC values are asymptotically normally distributed, but calculating their standard error (SE) is non-trivial when f is not a linear regression model.[12,20]

ShapleyVIC definition

Our proposed ShapleyVIC is a hybrid of ante hoc and post hoc approaches, where the MR for each model in the Rashomon set is based on SAGE values. In the presence of collinearity among variables, we hypothesize that negative SAGE values with large absolute values are artifacts induced by highly correlated variables rather than indications of unimportance. Therefore, we define the Shapley-based MR based on the variance inflation factor[22] (VIF) of each variable:

$$mr_j^s(f) = \begin{cases} |\varphi_j(v_f)| \text{ if } VIF_j > v, \\ \varphi_j(v_f) \text{ if } VIF_j \le v, \end{cases}$$

where $j = 1, \ldots, d$, the superscript s indicates the Shapley-based approach, and v is a threshold for strong correlation. In our experiments, we used $v = 2$. Colinear variables will have similar MR values. The corresponding ShapleyVIC is:

$$VIC^S(R) = \{MR^S(f) : f(X_D) \in R(\varepsilon, f^*, F)\},$$

where $MR^S(f) = \{mr_1{}^s(f), \ldots, mr_d{}^s(f)\}$.

Pooling ShapleyVIC values using random effects meta-analysis

With the Shapley-based MR of each variable, we pool the values across the M nearly optimal models to assess the overall importance of each variable using a meta-analysis approach, viewing each model as a separate study. We denote the ShapleyVIC value of the j-th variable for the m-th model and its variance (estimated from the SAGE algorithm) by mr_{jm}^s and σ_{jm}^2, respectively. To simplify notation, we drop the subscript j in the rest of this subsection. Let θ_m denote the true ShapleyVIC value of this variable for the m-th model, where $mr_m^s \sim N(\theta_m, \sigma_m^2)$. Since different models have different coefficients for variables and therefore different levels of reliance on each variable, θ_m is expected to differ across models. Hence, we adopt the random effects approach in meta-analysis[23–25] and assume a normal distribution for the true MR, $\theta_m \sim N(\theta, \tau^2)$, where the grand mean across models, θ, and the between-model variability, τ^2, are to be estimated.

We estimate τ^2 using the commonly used DerSimonian-Laird approach.[23,25] The between-model variability (τ^2) and within-model variability (σ_m^2, $m = 1, \ldots, M$, estimated from SAGE) are two sources of the total variance (Q), which is the weighted average of the squared deviation of mr_m^s from its weighted average: $Q = \sum w_m \{mr_m^s - (\sum w_m mr_m^s)/(\sum w_m)\}^2$, with $w_m = 1/\sigma_m^2$. When within-model variability is the only source of total variance, Q is expected to be $M-1$. Hence, when $Q > M-1$, the between-model variance can be estimated by $\tau^2 = (Q-(M-1))/C$, where $C = (\sum w_m) - (\sum w_m^2)/(\sum w_m)$ is a scaling constant. If $Q \le M-1$, the estimated between-model variance is simply $\tau^2 = 0$.

With the estimated between-model variance, τ^2, the grand mean, θ, is estimated by a weighted average of mr_m^s: $\overline{mr}^s = (\sum w'_m mr_m^s)/(\sum w'_m)$ and $var(\overline{mr}^s) = 1/(\sum w'_m)$, where $w'_m = 1/(\sigma_m^2 + \tau^2)$.[23] The ShapleyVIC value from a new model within the Rashomon set, mr_{new}^s, may be predicted by assuming a t-distribution with $M-2$ degrees of freedom for $(mr_{new}^s - \overline{mr}^s)/\sqrt{(var(\overline{mr}^s) + \tau^2)}$.[24] The 95% prediction interval (PI) for mr_{new}^s is hence the 2.5th and 97.5th percentiles of this t-distribution.

ShapleyVIC inference

Only positive ShapleyVIC values indicate importance, and larger values suggest higher importance. A desirable property of ShapleyVIC is that the SE of each value is readily available from the SAGE algorithm: $\sigma_j(f) = SE(\widehat{mr}_j^s(f)) = SE(\widehat{\varphi}_j(v_f))$. This allows us to easily compare the reliance of a model on any two variables,

Table 1. Summary statistics of the 6 variables in the COMPAS study

Variable n (%)	All (n = 7,214)	No 2-year recidivism (n = 3,743)	With 2-year recidivism (n = 3,471)	Chi-squared test p value
Age				
18–20 years	220 (3.0)	47 (1.3)	173 (5.0)	<0.001
>20 years	6,994 (97.0)	3,696 (98.7)	3,298 (95.0)	
Gender				
Female	1,395 (19.3)	865 (23.1)	530 (15.3)	<0.001
Male	5,819 (80.7)	2,878 (76.9)	2,941 (84.7)	
Race				
African American	3,696 (51.2)	1,660 (44.3)	2,036 (58.7)	<0.001
Others	3,518 (48.8)	2,083 (55.7)	1,435 (41.3)	
Prior criminal history				
Yes	2,150 (29.8)	1,478 (39.5)	672 (19.4)	<0.001
No	5,064 (70.2)	2,265 (60.5)	2,799 (80.6)	
Juvenile criminal history				
Yes	6,241 (86.5)	3,489 (93.2)	2,752 (79.3)	<0.001
No	973 (13.5)	254 (6.8)	719 (20.7)	
Current charge				
Degree misdemeanor	2,548 (35.3)	1,496 (40.0)	1,052 (30.3)	<0.001
Others	4,666 (64.7)	2,247 (60.0)	2,419 (69.7)	

$\{X_j, X_k\} \in X_D$, where the difference is normally distributed with variance $var\{\widehat{mr}_j^s(f) - \widehat{mr}_k^s(f)\} = \sigma_j^2(f) + \sigma_k^2(f)$ (assuming independence between $mr_j^s(f)$ and $mr_k^s(f)$). The importance of the d variables to the model can be ranked based on the number of times each variable has significantly larger ShapleyVIC value than the other $d-1$ variables.

As described in the previous section, the average ShapleyVIC value of a variable indicates its overall importance across nearly optimal models, and the 95% PI for a new model from the Rashomon set can be used to statistically assess and compare overall importance. Since only positive values indicate importance, the overall importance of a variable is only statistically significant when the lower bound of the 95% PI is positive. We visualize the average ShapleyVIC value and the 95% PI using a bar plot with error bars and complement it with a colored violin plot of the distribution of MR and its relationship with model performance. Our proposed visualizations and their interpretations will be described in our empirical experiments.

ShapleyVIC implementation

Although VIC is validly computed from the same data used to train the optimal model,[20] we adopt the approach in SHAP and SAGE[16,26] to evaluate ShapleyVIC values using the test set and use the training set to train the optimal model and identify the Rashomon set. A larger sample requires a longer computation time;[16,27] therefore, we do not recommend using test sets larger than necessary for the algorithm to converge.

As a hybrid of model-agnostic VIC and SAGE, ShapleyVIC is also model agnostic. In view of the popularity of scoring models, which are often built upon regression models, in this paper, we focus on the implementation of ShapleyVIC with regression models. In such scenarios, the Rashomon set consists of regression coefficients, $\boldsymbol{\beta}$, corresponding to expected loss $E\{L\} \leq (1+\varepsilon) E\{L^*\}$, where the superscript asterisk (*) indicates the optimal model with minimum expected loss, and $\varepsilon = 5\%$ is an acceptable value. To generate a reasonable sample of $\boldsymbol{\beta}$, we consider a pragmatic sampling approach based on rejection sampling:

- Set initial values for M_0 (the number of initial samples to draw from the Rashomon set), and u_1 and u_2 (bounds of a uniform distribution).
- For each $i = 1, \ldots, M_0$, generate $k_i \sim U(u_1, u_2)$.
- Draw the i-th sample from a multivariate normal distribution: $\boldsymbol{\beta}_i \sim N(\boldsymbol{\beta}^*, k_i\Sigma^*)$, where $\boldsymbol{\beta}^*$ is the regression coefficients of the optimal model, and Σ^* is its variance-covariance matrix. Reject $\boldsymbol{\beta}_i$ if the corresponding empirical loss, $\widehat{L}_i$, exceeds the upper bound, i.e., if $\widehat{L}_i > (1+\varepsilon)\widehat{L}^*$.
- Adjust the values of M_0, u_1, and u_2 such that the range between $\widehat{L}^*$ and $(1+\varepsilon)\widehat{L}^*$ is well represented.

Advice on how to tune parameters M_0, u_1, and u_2 based on our empirical experiments is provided in Experimental procedures. Following the practice of Dong and Rudin,[20] we randomly selected a final sample of 300–400 models. We implemented ShapleyVIC as an R package, which is available from https://github.com/nliulab/ShapleyVIC.

When working with logistic regression models, Dong and Rudin[20] sampled $\boldsymbol{\beta}$ via an ellipsoid approximation to the Rashomon set, which worked well in their examples. When working with data with strong collinearity (e.g., see experiment 2 in the next section), however, we found it easier to explore a wide range of $\boldsymbol{\beta}$ using our sampling approach than using the ellipsoid approximation. Hence, we find our sampling approach a reasonable alternative to Dong and Rudin's approach for exploring the variability in variable contributions, which favors a wider coverage in the Rashomon space.

Experimental results

We used two data examples to demonstrate the implementation of ShapleyVIC and describe our proposed visualizations. In the first experiment, we motivated and validated ShapleyVIC by reproducing key findings in the recidivism prediction study of Dong and Rudin,[20] where the analysis of nearly optimal models suggested an overclaim of variable importance based on the optimal model. The second example analyzed electronic health records data with a higher dimension and a strong correlation. Moreover, we used the two examples to illustrate the use of ShapleyVIC as a complement to the SHAP analysis. In our proposed SHAP-ShapleyVIC framework, we assess variable contributions first using a conventional SHAP analysis of the optimal model and, next, with a ShapleyVIC assessment of nearly optimal models for additional insights. As detailed below, the SHAP-ShapleyVIC framework enables the interpretation of models on various levels, ranging from variable contributions to individual instances to the significance of overall importance across well-performing models, which are not simultaneously available from other IML approaches.

Experiment 1: Recidivism prediction study

This study aimed to assess the importance of six binary variables for predicting 2-year recidivism: age (dichotomized at 20 years),

CellPress
OPEN ACCESS

A

Optimal logistic regression model with minimum expected loss.

Variables	Estimated coefficients	Standard error	P-value	VIF
Intercept	0.445	0.107	<0.001	--
Prior conviction	-0.854	0.061	<0.001	1.06
History of juvenile crime	-0.865	0.084	<0.001	1.04
Age	1.500	0.187	<0.001	1.03
Race	0.416	0.053	<0.001	1.02
Current charge	-0.254	0.056	<0.001	1.02
Gender	0.384	0.068	<0.001	1.01

SHAP analysis of the optimal model

B Variable ranking based on optimal model.

C Variable contributions to optimal model.

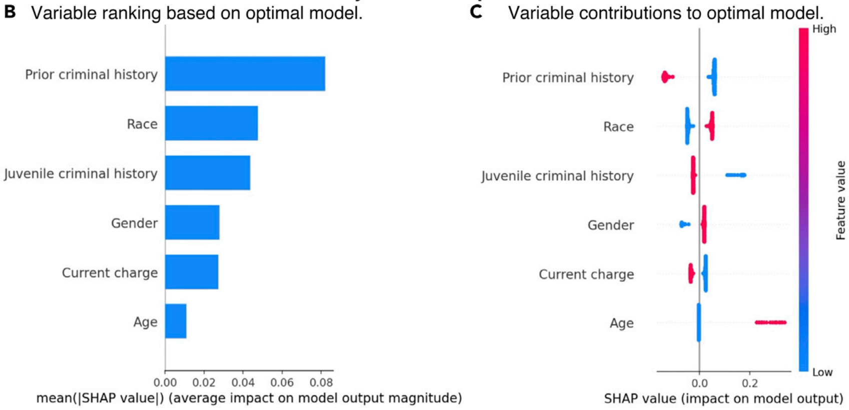

Figure 1. Visual summary of recidivism prediction study results from SHAP-ShapleyVIC framework, part I: SHAP analysis of the optimal model

(A) The optimal logistic regression model, where low variance inflation factors (VIF close to 1) did not suggest strong correlation.

(B) Variable ranking based on mean absolute SHAP values from the optimal model.

(C) SHAP values (represented by dots) indicate variable contributions to individual predictions.

race (African American or others), having prior criminal history, having juvenile criminal history, and current charge (degree misdemeanor or others), with a particular interest in race. The data include 7,214 records, and we pre-processed the data using code shared by Dong and Rudin[28] (see Table 1 for summary statistics). We randomly divided the data into a training set with 90% (6,393) of the records and a test set with the other 10% (721) and generated 350 nearly optimal logistic regression models.

SHAP analysis of optimal model. With only six variables and mild correlation among variables (VIF < 1.1 for all variables based on the optimal model; see Figure 1A), the optimal model is straightforward to interpret: controlling for other factors, African Americans had a higher risk of 2-year recidivism than other race groups. The SHAP analysis made the importance of race to the optimal model more explicit: it was the second most important variable based on the mean absolute SHAP values (see Figure 1B), with lower importance than prior criminal history and similar importance as juvenile criminal history, and the two race groups had a similar magnitude of impact on the outcome but in the opposite direction (see Figure 1C). Unlike SAGE, variances of SHAP values are not easily available for statistical assessments.

ShapleyVIC analysis of nearly optimal models and proposed visualizations. While race was found to be important to the optimal model in the SHAP analysis, whether it is important to the general prediction of 2-year recidivism requires further investigation of nearly optimal models. By analyzing 350 nearly optimal models using ShapleyVIC, we present a less biased assessment on variable importance.

In view of the small VIF values for all variables, the ShapleyVIC values were based on unadjusted SAGE values. We first assessed the overall importance of race by inspecting the bar plot of the average ShapleyVIC values (with 95% PI) across the 350 models (see Figure 2A). A small negative average MR (indicated by the bar) and a 95% PI containing zero indicated a non-significant overall importance to race, as opposed to the high importance based on the optimal model. This is consistent with the finding from Dong and Rudin[20] that, generally, race is not an important predictor of 2-year recidivism. Similarly, gender and current charge also had non-significant overall importance, indicated by the 95% PI being entirely below zero. Juvenile and prior criminal history were now ranked top with similar levels of overall importance that were significantly higher than those of the other four variables. Age, which was least important to the optimal model, had a moderate yet significant overall importance.

Inference on the bar plot of overall importance alone may lead to a misperception that variable ranking is static. We convey the variability of variable importance across models by visualizing the relationship between MR on each variable and model performance using a colored violin plot (see Figure 2B). The horizontal spread of a violin represents the range of MR on a variable, which is divided into slices of equal width. The height of each slice represents the proportion of models in the MR interval, and the color indicates the average performance (in terms of empirical loss) of these models. If an MR interval does not contain any model (which often occurs near the ends), the corresponding slice is combined with the neighbor closer to the center.

As illustrated by the well-mixed color across the range of each violin plot (see Figure 2B), there is no simple relationship between model performance and reliance on any variable, regardless of its overall variable importance. For race, most dark-colored strips in the violin plot are positioned at negative MR values, suggesting that better performing models tended to have a low reliance on race. Bar and violin plots of VIC values from the same 350 models (see Figure S1) suggested similar findings but without variability measures to statistically test and compare variable importance.

Experiment 2: MIMIC study

In this study, we examined the importance of 21 variables (including age, clinical tests, and vital signs; see Table 2 for a full variable list and summary statistics) in predicting 24-h mortality in intensive care units (ICUs) using a random sample of 20,000

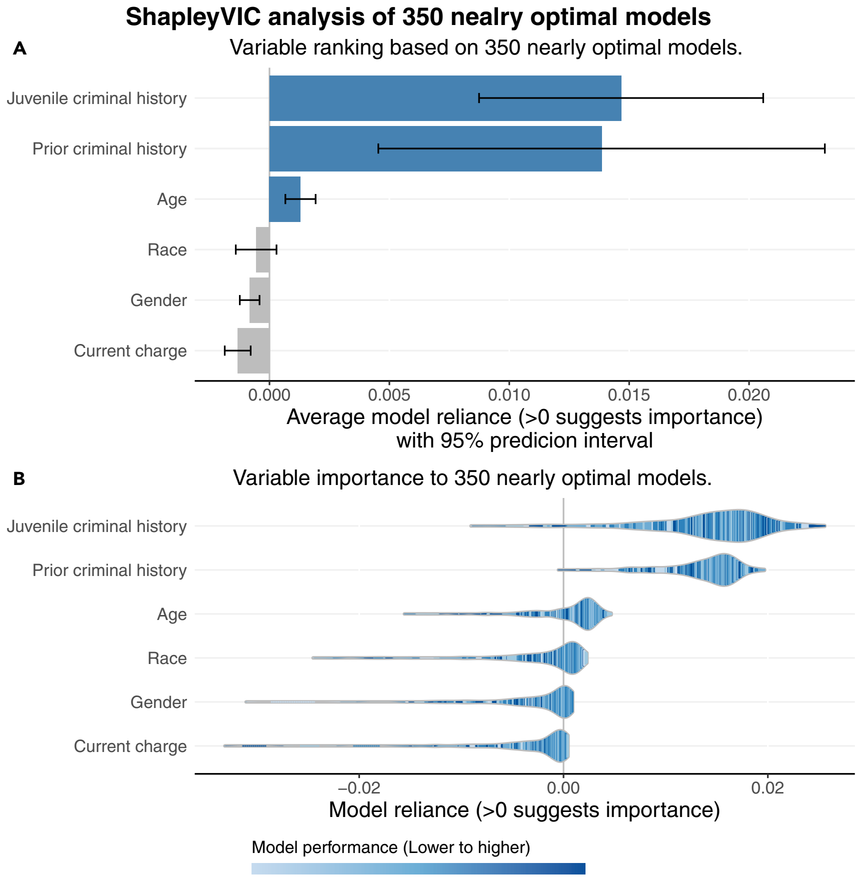

Figure 2. Visual summary of recidivism prediction study results from SHAP-ShapleyVIC framework, part II: ShapleyVIC analysis of nearly optimal models

(A) ShapleyVIC suggested non-significant overall importance for race after accounting for the variability in variable importance across the 350 nearly optimal models.

(B) Distribution of variable importance (indicated by the shape of violin plots) and the corresponding model performance (indicated by color) to complement inference on average ShapleyVIC values.

adult patients from the BIDMC dataset of the Medical Information Mart for Intensive Care (MIMIC) III database. We trained a logistic regression and generated a sample of 350 nearly optimal models using a random sample of 17,000 records and used the rest of the 3,000 records to evaluate variable importance.

SHAP analysis of optimal model. The extremely small p values (<0.001; see Figure 3A) for two-thirds of the variables and collinearity among variables (indicated by large VIF values in Figure 3A and strong correlations in Figure S2) made it difficult to rank variable importance based on the optimal model. SHAP analysis of the model enabled straightforward variable ranking using mean absolute SHAP values (see Figure 3B). Per-instance SHAP values (indicated by dots in Figure 3C) provided additional insights on variable contributions to the optimal model, e.g., although creatinine only ranked 14th among the 21 variables, high creatinine levels can have a strong impact on predictions. However, the statistical significance of such an impact is unknown.

ShapleyVIC analysis of nearly optimal models and proposed visualizations. SHAP analysis of the optimal model does not answer some practical question, e.g., is creatinine deemed to contribute moderately to general prediction of mortality using logistic regression? This is answered by the extended global interpretation using ShapleyVIC.

We found a threshold of VIF >2 identified all variables involved in moderate to strong correlations. The ShapleyVIC values for the 11 variables with VIF >2 were based on the absolute SAGE values, whereas for the other 10 variables, the unadjusted SAGE values were used. Figure 4A presents variable ranking after accounting for the variability in variable importance across the 350 nearly optimal models. Six of the top seven variables based on mean absolute SHAP values were also ranked top seven by average ShapleyVIC values, but ShapleyVIC tended to rank rest of the variables differently. The 95% PIs of average ShapleyVIC values suggested similar importance for the 5th- to 7th-ranking variables and statistically non-significant overall importance for the last five variables. We also used VIC to analyze the same 350 models and had similar findings as ShapleyVIC on top-ranking variables (see Figure S3).

As highlighted in experiment 1, it is important to inspect the variability of variable importance across models using the colored violin plot to avoid misperceptions. Generally, creatinine contributed significantly to nearly optimal models and ranked 13th based on the average ShapleyVIC value (see Figure 4A). However, the violin plot (see Figure 4B) showed a wide spread of ShapleyVIC values for creatinine across models, and the dark blue strip at the right end suggested the presence of well-performing models that relied heavily on creatinine. To extract models with heavy reliance on creatinine for further investigation, we assessed the variable ranking in each of the 350 models by pairwise comparison of ShapleyVIC values (visually summarized in Figure 5) and further inspected the ranking data to identify 19 models where creatinine ranked top seven. Among these 19 models, creatinine increased to the 6th-ranking variable (see Figure 6), and hemoglobin and hematocrit had lower ranks, while other variables were not much affected. Further studies on creatinine may draw additional samples from the Rashomon set that are close to these 19 models for closer investigation.

DISCUSSION

Uncertainty is drawing attention when interpreting ML models,[10,29] which is relevant when interpreting predictions or estimated effects (e.g., see Tomsett et al.,[30] Antorán et al.,[31]

CellPress
OPEN ACCESS

Table 2. Summary statistics of the 21 variables in the MIMIC study

Variables median (first and third quartiles)	All (n = 20,000)	Discharged alive (n = 18,259)	Mortality (n = 1,741)	Mann-Whitney test p value
Age	64.4 (52.1, 75.9)	63.8 (51.6, 75.3)	71.2 (59.0, 80.5)	<0.001
Heart rate (beats/min)	84.4 (74.7, 95.1)	84.0 (74.5, 94.5)	90.3 (77.1, 103.4)	<0.001
Systolic blood pressure (SBP; mm Hg)	116.5 (107.0, 129.2)	116.9 (107.4, 129.4)	111.5 (101.5, 126.6)	<0.001
Diastolic blood pressure (DBP; mm Hg)	60.0 (53.7, 67.2)	60.2 (53.9, 67.5)	57.5 (51.0, 65.0)	<0.001
Mean arterial pressure (MAP; mm Hg)	76.9 (70.6, 84.8)	77.1 (70.9, 85.0)	74.2 (67.5, 82.4)	<0.001
Respiration (breaths/min)	18.0 (15.9, 20.6)	17.8 (15.8, 20.4)	19.9 (17.2, 23.5)	<0.001
Temperature (°C)	36.8 (36.5, 37.2)	36.8 (36.5, 37.2)	36.8 (36.3, 37.3)	<0.001
Peripheral capillary oxygen saturation (SpO_2; %)	97.6 (96.2, 98.7)	97.6 (96.3, 98.7)	97.4 (95.6, 98.8)	<0.001
Glucose (mg/dL)	129.0 (111.3, 154.0)	128.2 (111.0, 152.5)	138.3 (116.0, 168.1)	<0.001
Anion gap (mEq/L)	13.5 (12.0, 16.0)	13.5 (12.0, 15.5)	15.5 (13.5, 18.5)	<0.001
Bicarbonate (mmol/L)	24.0 (21.5, 26.0)	24.0 (22.0, 26.5)	22.5 (19.0, 26.0)	<0.001
Creatinine (μmol/L)	0.9 (0.7, 1.4)	0.9 (0.7, 1.3)	1.2 (0.8, 2.1)	<0.001
Chloride (mEq/L)	105.0 (101.5, 108.0)	105.0 (101.5, 108.0)	104.0 (99.5, 108.5)	<0.001
Hematocrit (%)	32.4 (28.7, 36.4)	32.5 (28.8, 36.5)	30.9 (27.7, 35.1)	<0.001
Hemoglobin (g/dL)	10.9 (9.6, 12.3)	10.9 (9.7, 12.4)	10.3 (9.2, 11.7)	<0.001
Lactate (mmol/L)	1.8 (1.7, 2.0)	1.8 (1.7, 2.0)	1.8 (1.8, 3.3)	<0.001
Platelet (thousand per microliter)	209.0 (154.0, 277.0)	210.0 (156.5, 277.0)	194.0 (117.0, 282.0)	<0.001
Potassium (mmol/L)	4.2 (3.8, 4.5)	4.2 (3.8, 4.5)	4.2 (3.8, 4.6)	0.028
Blood urea nitrogen (BUN; mg/dL)	18.0 (12.5, 29.5)	17.5 (12.5, 27.5)	28.5 (18.0, 48.0)	<0.001
Sodium (mmol/L)	138.5 (136.0, 140.5)	138.5 (136.0, 140.5)	138.5 (135.0, 141.5)	0.715
White blood cells (WBCs; thousand per microliter)	10.8 (7.9, 14.2)	10.7 (7.9, 14.0)	12.4 (8.8, 17.1)	<0.001

and Ghosal and Tucker[32]) and when assessing the importance of variables (e.g., see Schwab and Karlen[33] and Fabi and Schneider[34]). Specifically, uncertainty of variable importance is relevant not only to causal interpretation of models[33] but also to the causability of model explanations, i.e., the ability of the explanation in conveying a specific level of causal understanding to a human expert.[35] We contribute to the investigation of uncertainty from a largely neglected source: the uncertainty in variable importance among nearly optimal models (e.g., where model loss is within an acceptable range) that could have been selected in a prediction task for practical considerations. By actively investigating the association between model performance and reliance on each variable, we provide a higher-level global assessment that studies model ensembles to avoid bias toward a single model when inferring variable importance (or unimportance) and provide a basis for building interpretable models under practical considerations and constraints.

The recently proposed VIC[20] is the first to demonstrate the benefit of extending global variable importance assessment to nearly optimal models. Our proposed method, named ShapleyVIC, is a hybrid of state-of-the-art ante hoc and post hoc IML approaches that extends the widely used Shapley-based explanations to global interpretations beyond a single optimal model. Using the meta-analysis approach, we pool the Shapley-based importance (measured by SAGE with uncertainty interval) from each model to explicitly quantify the uncertainty across models and summarize the overall importance of each variable. This allows us to support inference on variable importance with statistical evidence, which is not easily available from VIC.[20] The close connection between SHAP and SAGE[16,21] enables a seamless integration of ShapleyVIC with the state-of-the-art SHAP method for additional insight on variable contributions, enabling local and global interpretations as well as overall importance assessments across well-performing models that are not simultaneously available from other IML approaches. Our proposed visualizations effectively communicate different levels of information and work well for high-dimensional data.

Our empirical experiments demonstrate the application of our proposed SHAP-ShapleyVIC framework. SHAP analysis of the optimal model facilitates straightforward interpretation of variable contribution, and subsequent ShapleyVIC analysis of nearly optimal models updates the assessment by accounting for the variability in variable importance. Our experiment on recidivism prediction provides a strong motivation for extending global interpretation beyond a single model, where ShapleyVIC found that the importance of race in predicting recidivism in a post hoc assessment was likely a random noise. By identifying variables with similar overall importance based on the variability between models, ShapleyVIC adds flexibility to model-building steps, e.g., by considering the stepwise inclusion or exclusion of such variables. Using our proposed visualizations of ShapleyVIC values across models, we demonstrated in the MIMIC study how to identify the presence of models with higher reliance on a variable of interest and subsequently focus on the relevant subset of models for additional information.

A Optimal logistic regression model with minimum expected loss.

Variable	Estimated coefficient	Standard error	P-value	VIF
Intercept	3.504	2.314	0.130	--
Hemoglobin (g/dL)	-0.274	0.061	0.000	15.42
Hematocrit (%)	0.073	0.021	0.001	15.40
Chloride (mEq/L)	-0.043	0.018	0.020	15.27
Bicarbonate (mmol/L)	-0.033	0.019	0.094	10.26
Sodium (mmol/L)	0.026	0.019	0.167	9.80
Mean arterial pressure (MAP; mm Hg)	0.031	0.008	0.000	7.99
Anion gap (mEq/L)	0.052	0.021	0.012	7.44
Diastolic blood pressure (DBP; mm Hg)	-0.026	0.006	0.000	4.85
Systolic blood pressure (SBP; mm Hg)	-0.016	0.003	0.000	3.19
Creatinine (μmol/L)	-0.135	0.028	0.000	2.17
Blood urea nitrogen (BUN; mg/dL)	0.018	0.002	0.000	2.06
Heart rate (beats/min)	0.015	0.002	0.000	1.48
Potassium (mmol/L)	-0.172	0.053	0.001	1.34
Lactate (mmol/L)	0.236	0.021	0.000	1.32
Age (years)	0.024	0.002	0.000	1.27
Respiration (breaths/min)	0.074	0.007	0.000	1.24
Temperature (°C)	-0.109	0.051	0.032	1.20
Platelet (thousand per microliter)	-0.002	0.000	0.000	1.16
Peripheral capillary oxygen saturation (SpO_2; %)	-0.039	0.013	0.002	1.12
White blood cells (WBC; thousand per microliter)	0.016	0.003	0.000	1.08
Glucose (mg/dL)	0.001	0.001	0.191	1.07

SHAP analysis of the optimal model

B Variable ranking based on optimal model. **C** Variable contributions to optimal model.

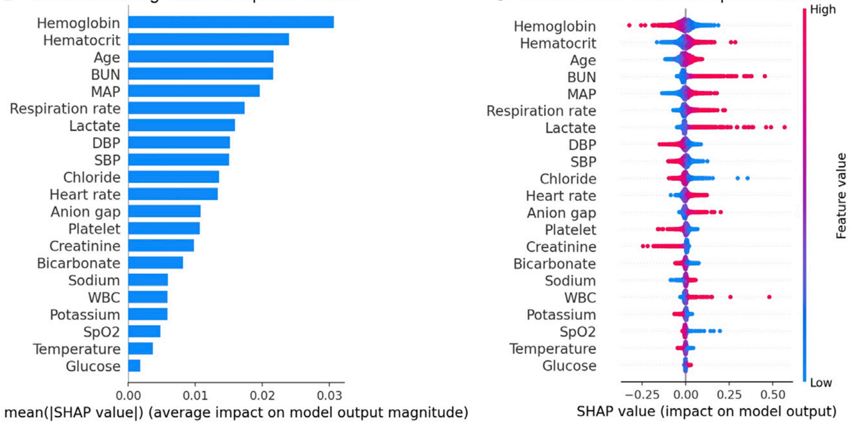

Figure 3. Visual summary of MIMIC study results from SHAP-ShapleyVIC framework, part I: SHAP analysis of the optimal model

(A) The optimal logistic regression model, where high variance inflation factors (VIF >2) suggested strong correlation for some variables (indicated by gray).

(B) Variable ranking based on mean absolute SHAP values from the optimal model.

(C) SHAP values (represented by dots) indicate variable contributions to individual predictions.

In common with VIC, ShapleyVIC faces a challenge in drawing representative samples of nearly optimal models due to the difficulty in characterizing the Rashomon set.[12,20,29] In our MIMIC study with strong collinearity, we found it easier to explore a wide range in the Rashomon set for some variables using our pragmatic sampling approach than by using the more disciplined ellipsoid approximation approach described by Dong and Rudin.[20] Our pragmatic sampling approach may not preserve the asymptotic properties based on the Rashomon set,[12,20] but by using the standard deviation of the Shapley-based MR, we are able to pool information across sampled models even when such asymptotic properties do not hold. In view of the renewed interest in developing inherently interpretable prediction models (e.g., the easy-to-interpret scoring models),[7] in this paper, we have focused on exploring Rashomon sets for regression models. Dong and Rudin[20] described an algorithm for sampling the Rashomon set of decision trees, and future work should develop sampling algorithms for general ML models for broader applications. However, it is worth noting that such practical challenges in generating nearly optimal models does not invalidate the theoretical model-agnostic property of ShapleyVIC values.

Our meta-analysis approach for pooling ShapleyVIC values assumes normality, which affects the PIs but less so for the average values.[36] This may be an issue for some variables in our experiments (e.g., the anion gap that had a bimodal distribution and the few variables with extreme left tails and negative average values) but is not likely to invalidate our assessment on the overall importance of affected variables given the range of their ShapleyVIC values and estimated averages. Future work will consider alternative meta-analysis approaches with less restrictive assumptions.[36,37] In addition to comparing average ShapleyVIC values, we also ranked variables based on t-test comparisons of ShapleyVIC values between all variable pairs for each model and used the ranking to filter for models of interest. Future work can explore for alternative methods to statistically compare variable importance within models, investigate the variability in ranking across models, and discuss the practical implications.

Strong correlation among variables (e.g., in the MIMIC study) also poses a challenge on variable importance assessments. Permutation importance is susceptible to biases when applied to correlated data, as it samples from the marginal distribution.[16] SAGE is defined using the conditional distribution to account for correlations, but due to the immense computational challenge, the authors adopted a sampling-based approximation approach that generates variables from marginal distributions and consequently assumes some extent of independence.[16] Similar challenges are encountered by other practical implementations of Shapley-based methods (e.g., see Covert et al.[21,38]) and are not easily resolved. By using the absolute value of SAGE as a measure of MR for highly correlated variables, measured by VIF, we provide a pragmatic solution to this problem that may inspire a more disciplined solution. Although VIF >2 worked well in both data examples, its generalizability to other data remains to be investigated. ShapleyVIC may also be used with other (global) variable importance measures for preferrable properties.

In conclusion, in this study we present ShapleyVIC, a hybrid of the state-of-the-art ante hoc and post hoc IML approaches, that comprehensively assesses variable importance by extending the investigation to nearly optimal models that are relevant to practical prediction tasks. ShapleyVIC seamlessly integrates with SHAP due to a common theoretical basis, extending current IML applications to global interpretations and beyond. Although

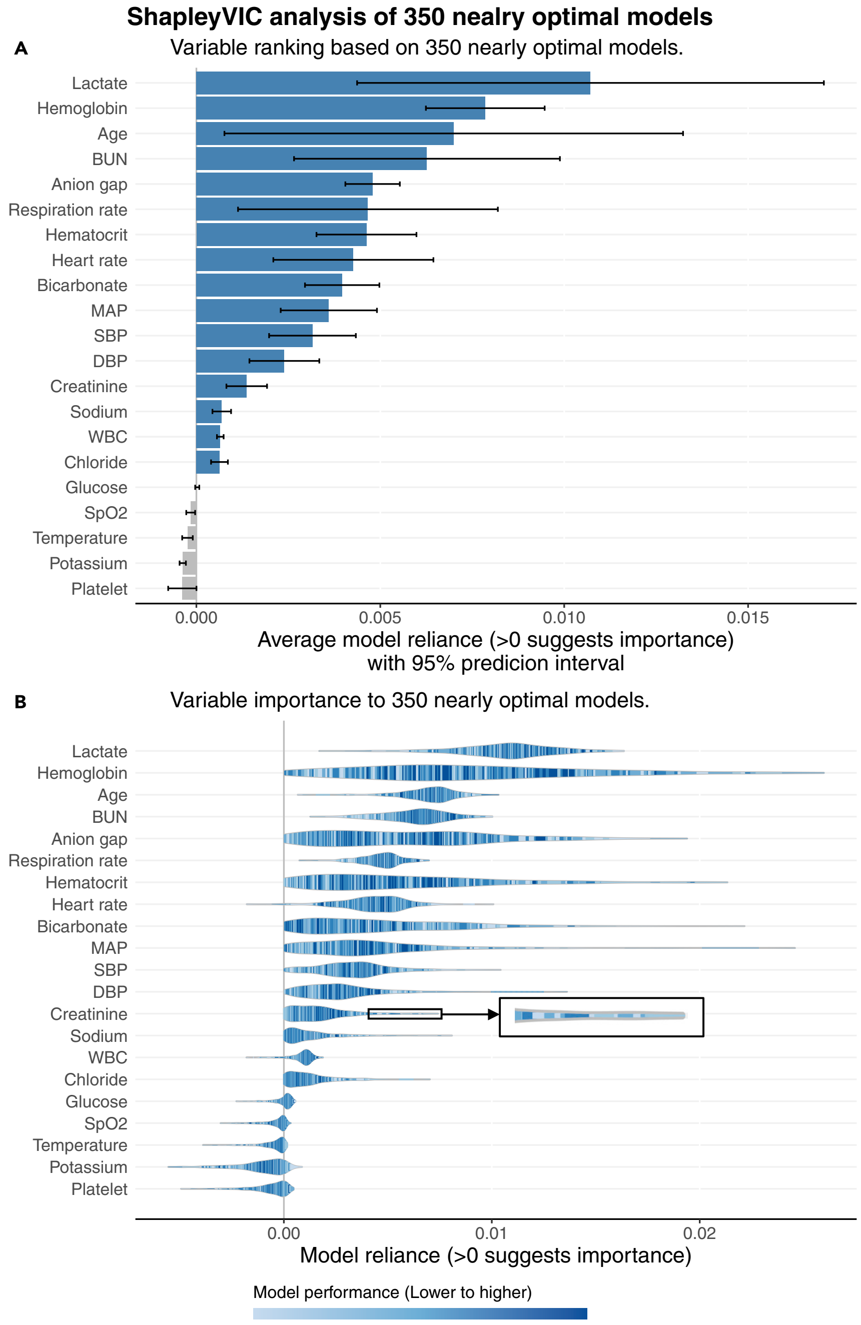

Figure 4. Visual summary of MIMIC study results from SHAP-ShapleyVIC framework, part II: ShapleyVIC analysis of nearly optimal models

(A) ShapleyVIC suggested a different variable ranking after accounting for the variability in variable importance across the 350 nearly optimal models.

(B) Distribution of variable importance (indicated by the shape of violin plots) and the corresponding model performance (indicated by color) to complement inference on average ShapleyVIC values. Dark blue strips towards the right end of a violin plot suggests the presence of good models that relied heavily on the variable (e.g., creatinine) for further investigations.

we described the implementation of ShapleyVIC with simple regression models, which can be readily integrated with the development of scoring models (e.g., the recently developed AutoScore framework[19]), ShapleyVIC is model-agnostic and applicable for other ML models.

EXPERIMENTAL PROCEDURES

Resource availability

Lead contact

Further information and requests for resources should be directed to and will be fulfilled by the lead contact, Nan Liu (liu.nan@duke-nus.edu.sg).

Materials availability

This research did not generate any materials.

Data and code availability

The MIMIC data are publicly available subject to the completion of ethics training and a signed data use agreement and are for research only. The recidivism prediction data and all original code have been deposited at Zenodo under https://doi.org/10.5281/zenodo.5904414 and are publicly available as of the date of publication.

Tuning parameters for sampling the Rashomon set

We advise first tuning parameters u_1 and u_2 using a temporary value for M_0 that is smaller than necessary for the final sample (e.g., M_0 = 200) to reduce run time. Researchers may begin with values u_1 = 0.5 and u_2 = 1 to generate M_0

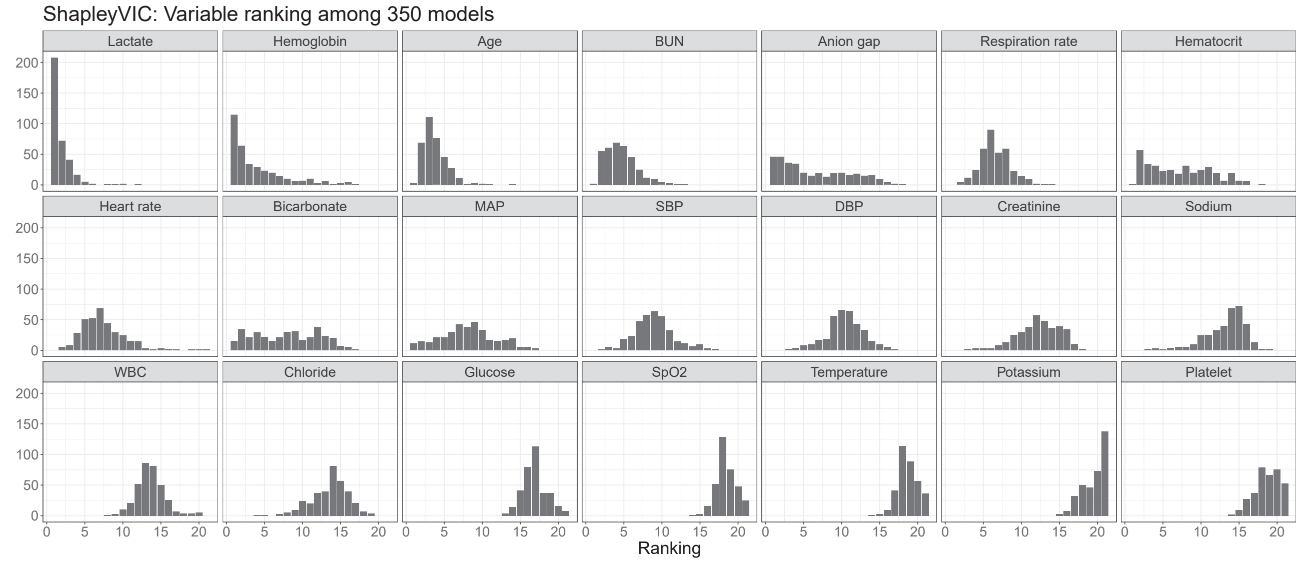

Figure 5. Frequency of ranking of each variable in the MIMIC study based on pairwise comparison of model reliance
Variables are arranged by average ShapleyVIC values.

samples of regression coefficients, compute the corresponding empirical loss, inspect the range of empirical loss, and count the number of models not rejected. These steps normally take less than a minute for given values of u_1 and u_2. Based on our two experiments, it may suffice to keep $u_1 = 0.5$ and adjust u_2 until the range of loss in the Rashomon set is well represented. In view of the large size of the training data, the initial choice of $u_2 = 1$ is likely too small to fully explore the Rashomon set, resulting in all models being accepted and the corresponding empirical loss being very close to the minimum loss. In our two experiments, we incremented the values for u_2 by 10 to speed up the tuning process and eventually selected values 80 and 20 for u_1 and u_2, respectively. Finally, given the selected values for u_1 and u_2 and the number of samples kept given the initial choice of M_0, researchers can increase the value for M_0 (e.g., to 800 in both experiments) to obtain a reasonable number of final samples.

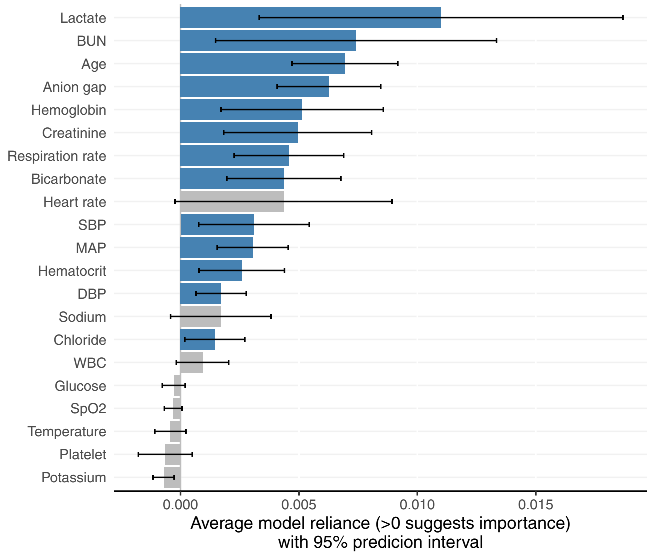

Figure 6. Bar plot of average ShapleyVIC values from 19 models where creatinine ranked top 7

SUPPLEMENTAL INFORMATION

Supplemental information can be found online at https://doi.org/10.1016/j.patter.2022.100452.

ACKNOWLEDGMENTS

Y.N. is supported by the Khoo Postdoctoral Fellowship Award (project no. Duke-NUS-KPFA/2021/0051) from the Estate of Tan Sri Khoo Teck Puat.

AUTHOR CONTRIBUTIONS

N.L. and Y.N. conceptualized the study and designed the analytical method. Y.N. developed the software package for the method, conducted data analyses, and drafted the manuscript. All authors interpreted the data, discussed the results, and critically revised the manuscript for intellectual content. N.L. supervised the study.

DECLARATION OF INTERESTS

The authors declare no competing interests.

Received: October 19, 2021
Revised: December 28, 2021
Accepted: January 27, 2022
Published: February 22, 2022

REFERENCES

1. Ting, D.S.W., Cheung, C.Y.L., Lim, G., Tan, G.S.W., Quang, N.D., Gan, A., Hamzah, H., Garcia-Franco, R., Yeo, I.Y.S., Lee, S.Y., et al. (2017). Development and validation of a deep learning system for diabetic retinopathy and related eye diseases using retinal images from multiethnic populations with diabetes. JAMA *318*, 2211–2223. https://doi.org/10.1001/jama.2017.18152.
2. Xie, Y., Nguyen, Q.D., Hamzah, H., Lim, G., Bellemo, V., Gunasekeran, D.V., Yip, M.Y.T., Lee, X.Q., Hsu, W., Lee, M.L., et al. (2020). Artificial intelligence for teleophthalmology-based diabetic retinopathy screening in a national programme: an economic analysis modelling study. Lancet Digit. Heal. *2*, e240–e249. https://doi.org/10.1016/S2589-7500(20)30060-1.

CellPress
OPEN ACCESS

3. Stiglic, G., Kocbek, P., Fijacko, N., Zitnik, M., Verbert, K., and Cilar, L. (2020). Interpretability of machine learning-based prediction models in healthcare. Wires Data Min. Knowl. Discov. *10*, e1379. https://doi.org/10.1002/widm.1379.
4. Nassar, M., Salah, K., ur Rehman, M.H., and Svetinovic, D. (2020). Blockchain for explainable and trustworthy artificial intelligence. Wires Data Min. Knowl. Discov. *10*, e1340. https://doi.org/10.1002/widm.1340.
5. Emmert-Streib, F., Yli-Harja, O., and Dehmer, M. (2020). Explainable artificial intelligence and machine learning: a reality rooted perspective. Wires Data Min. Knowl. Discov. *10*, e1368. https://doi.org/10.1002/widm.1368.
6. Confalonieri, R., Coba, L., Wagner, B., and Besold, T.R. (2021). A historical perspective of explainable artificial intelligence. Wires Data Min. Knowl. Discov. *11*, e1391. https://doi.org/10.1002/widm.1391.
7. Rudin, C. (2019). Stop explaining black box machine learning models for high stakes decisions and use interpretable models instead. Nat. Mach. Intell. *1*, 206–215. https://doi.org/10.1038/s42256-019-0048-x.
8. Murdoch, W.J., Singh, C., Kumbier, K., Abbasi-Asl, R., and Yu, B. (2019). Definitions, methods, and applications in interpretable machine learning. Proc. Natl. Acad. Sci. *116*, 22071–22080. https://doi.org/10.1073/pnas.1900654116.
9. Arrieta, A.B., Díaz-Rodríguez, N., Del Ser, J., Bennetot, A., Tabik, S., Barbado, A., Garcia, S., Gil-Lopez, S., Molina, D., Benjamins, R., et al. (2020). Explainable artificial intelligence (XAI): concepts, taxonomies, opportunities and challenges toward responsible AI. Inf. Fusion *58*, 82–115. https://doi.org/10.1016/j.inffus.2019.12.012.
10. Molnar, C., Casalicchio, G., and Bischl, B. (2020). Interpretable machine learning – a brief history, state-of-the-art and challenges. In ECML PKDD 2020 Workshops. ECML PKDD 2020. Communications in Computer and Information Science, *1323*, I. Koprinska, ed. *(Cham: Springer)*, pp. 417–431. https://doi.org/10.1007/978-3-030-65965-3_28.
11. Breiman, L. (2001). Random forests. Mach. Learn. *45*, 5–32. https://doi.org/10.1023/A:1010933404324.
12. Fisher, A., Rudin, C., and Dominici, F. (2019). All models are wrong, but many are useful: learning a variable's importance by studying an entire class of prediction models simultaneously. J. Mach. Learn. Res. *20*, 1–81.
13. Linardatos, P., Papastefanopoulos, V., and Kotsiantis, S. (2021). Explainable AI: a review of machine learning interpretability methods. Entropy *23*, 18. https://doi.org/10.3390/e23010018.
14. Ribeiro, M.T., Singh, S., and Guestrin, C. (2016). "Why should i trust you?" Explaining the predictions of any classifier. In KDD '16: Proceedings of the 22nd ACM SIGKDD International Conference on Knowledge Discovery and Data Mining, B. Krishnapuram, M. Shah, A. Smola, C. Aggarwal, D. Shen, and R. Rastogi, eds. (Association for Computing Machinery), pp. 1135–1144.
15. Lundberg, S.M., and Lee, S.-I. (2017). A unified approach to interpreting model predictions. In Proceedings of the 31st International Conference on Neural Information Processing Systems, U. von Luxburg, I. Guyon, S. Bengio, H. Wallach, and R. Fergus, eds. (Curran Associates Inc.), pp. 4768–4777.
16. Covert, I.C., Lundberg, S., and Lee, S.-I. (2020). Understanding global feature contributions with additive importance measures. In Advances in Neural Information Processing Systems 33 (NeurIPS 2020), *33*, H. Larochelle, M. Ranzato, R. Hadsell, M.F. Balcan, and H. Lin, eds. (Curran Associates), pp. 17212–17223.
17. Ustun, B., and Rudin, C. (2016). Supersparse linear integer models for optimized medical scoring systems. Mach. Learn. *102*, 349–391. https://doi.org/10.1007/s10994-015-5528-6.
18. Ustun, B., and Rudin, C. (2019). Learning optimized risk scores. J. Mach. Learn. Res. *20*, 1–75.
19. Xie, F., Chakraborty, B., Ong, M.E.H., Goldstein, B.A., and Liu, N. (2020). AutoScore: a machine learning–based automatic clinical score generator and its application to mortality prediction using electronic health records. JMIR Med. Inform. *8*, e21798. https://doi.org/10.2196/21798.
20. Dong, J., and Rudin, C. (2020). Exploring the cloud of variable importance for the set of all good models. Nat. Mach. Intell. *2*, 810–824. https://doi.org/10.1038/s42256-020-00264-0.
21. Covert, I., Lundberg, S., and Lee, S.-I. (2020). Feature removal is a unifying principle for model explanation methods, Preprint at arXiv:2011.03623 https://arxiv.org/abs/2011.03623.
22. James, G., Witten, D., Hastie, T., and Tibshirani, R. (2021). An Introduction to Statistical Learning: With Applications in R, 2nd ed. (Springer US), pp. 99–103.
23. Borenstein, M., Hedges, L.V., Higgins, J.P.T., and Rothstein, H.R. (2010). A basic introduction to fixed-effect and random-effects models for meta-analysis. Res. Synth. Methods *1*, 97–111. https://doi.org/10.1002/jrsm.12.
24. Higgins, J.P.T., Thompson, S.G., and Spiegelhalter, D.J. (2009). A re-evaluation of random-effects meta-analysis. J. R. Stat. Soc. A. *172*, 137–159.
25. DerSimonian, R., and Laird, N. (1986). Meta-analysis in clinical trials. Control Clin. Trials *7*, 177–188. https://doi.org/10.1016/0197-2456(86)90046-2.
26. Lundberg, S.M., Erion, G., Chen, H., DeGrave, A., Prutkin, J.M., Nair, B., Katz, R., Himmelfarb, J., Bansal, N., and Lee, S.-I. (2020). From local explanations to global understanding with explainable AI for trees. Nat. Mach. Intell. *2*, 56–67. https://doi.org/10.1038/s42256-019-0138-9.
27. iancovert/sage: For calculating global feature importance using Shapley values. https://github.com/iancovert/sage.
28. Dong, J. (2020). Jiayun-Dong/vic v1.0.0 (v1.0.0) (Zenodo). https://doi.org/10.5281/zenodo.4065582.
29. Rudin, C., Chen, C., Chen, Z., Huang, H., Semenova, L., and Zhong, C. (2022). Interpretable machine learning: fundamental principles and 10 grand challenges. Stat. Surv. *16*, 1–85. https://doi.org/10.1214/21-SS133.
30. Tomsett, R., Preece, A., Braines, D., Cerutti, F., Chakraborty, S., Srivastava, M., Pearson, G., and Kaplan, L. (2020). Rapid trust calibration through interpretable and uncertainty-aware AI. Patterns *1*, 100049. https://doi.org/10.1016/j.patter.2020.100049.
31. Antorán, J., Bhatt, U., Adel, T., Weller, A., and Hernández-Lobato, J.M. (2020). Getting a CLUE: a method for explaining uncertainty estimates, Preprint at arXiv:2006.06848 https://arxiv.org/abs/2006.06848.
32. Ghoshal, B., and Tucker, A. (2020). Estimating uncertainty and interpretability in deep learning for coronavirus (COVID-19) detection, Preprint at arXiv:2003.10769 https://arxiv.org/abs/2003.10769.
33. Schwab, P., and Karlen, W. (2019). CXPlain: causal explanations for model interpretation under uncertainty. In Advances in Neural Information Processing Systems 32 (NeurIPS 2019), H. Wallach, H. Larochelle, A. Beygelzimer, F. d'Alché-Buc, E. Fox, and R. Garnett, eds. (Curran Associates Inc.), pp. 10220–10230.
34. Fabi, K., and Schneider, J. (2020). On feature relevance uncertainty: a Monte Carlo dropout sampling approach, Preprint at arXiv:2008.01468 https://arxiv.org/abs/2008.01468.
35. Holzinger, A., Malle, B., Saranti, A., and Pfeifer, B. (2021). Towards multi-modal causability with graph neural networks enabling information fusion for explainable AI. Inf. Fusion *71*, 28–37. https://doi.org/10.1016/j.inffus.2021.01.008.
36. Jackson, D., and White, I.R. (2018). When should meta-analysis avoid making hidden normality assumptions? Biometrical J. *60*, 1040–1058. https://doi.org/10.1002/bimj.201800071.
37. Veroniki, A.A., Jackson, D., Viechtbauer, W., Bender, R., Bowden, J., Knapp, G., Kuss, O., Higgins, J.P., Langan, D., and Salanti, G. (2016). Methods to estimate the between-study variance and its uncertainty in meta-analysis. Res. Synth. Methods *7*, 55–79. https://doi.org/10.1002/jrsm.1164.
38. Covert, I., and Lee, S.-I. (2021). Improving KernelSHAP: Practical Shapley Value Estimation Using Linear Regression. In Proceedings of The 24th International Conference on Artificial Intelligence and Statistics, A. Banerjee and K. Fukumizu, eds. (PMLR), pp. 3457–3465.

Patterns, Volume *3*

Supplemental information

Shapley variable importance cloud

for interpretable machine learning

Yilin Ning, Marcus Eng Hock Ong, Bibhas Chakraborty, Benjamin Alan Goldstein, Daniel Shu Wei Ting, Roger Vaughan, and Nan Liu

Figure S1. Visual summary of recidivism prediction study results from the VIC analysis. VIC values were subtracted 1 such that zero value or lower indicate unimportance. (A) Average values from the 350 nearly optimal models. Variable ranking was similar to that based on average ShapleyVIC values. (B) Distribution of VIC values (subtracted 1) and the corresponding model performance.

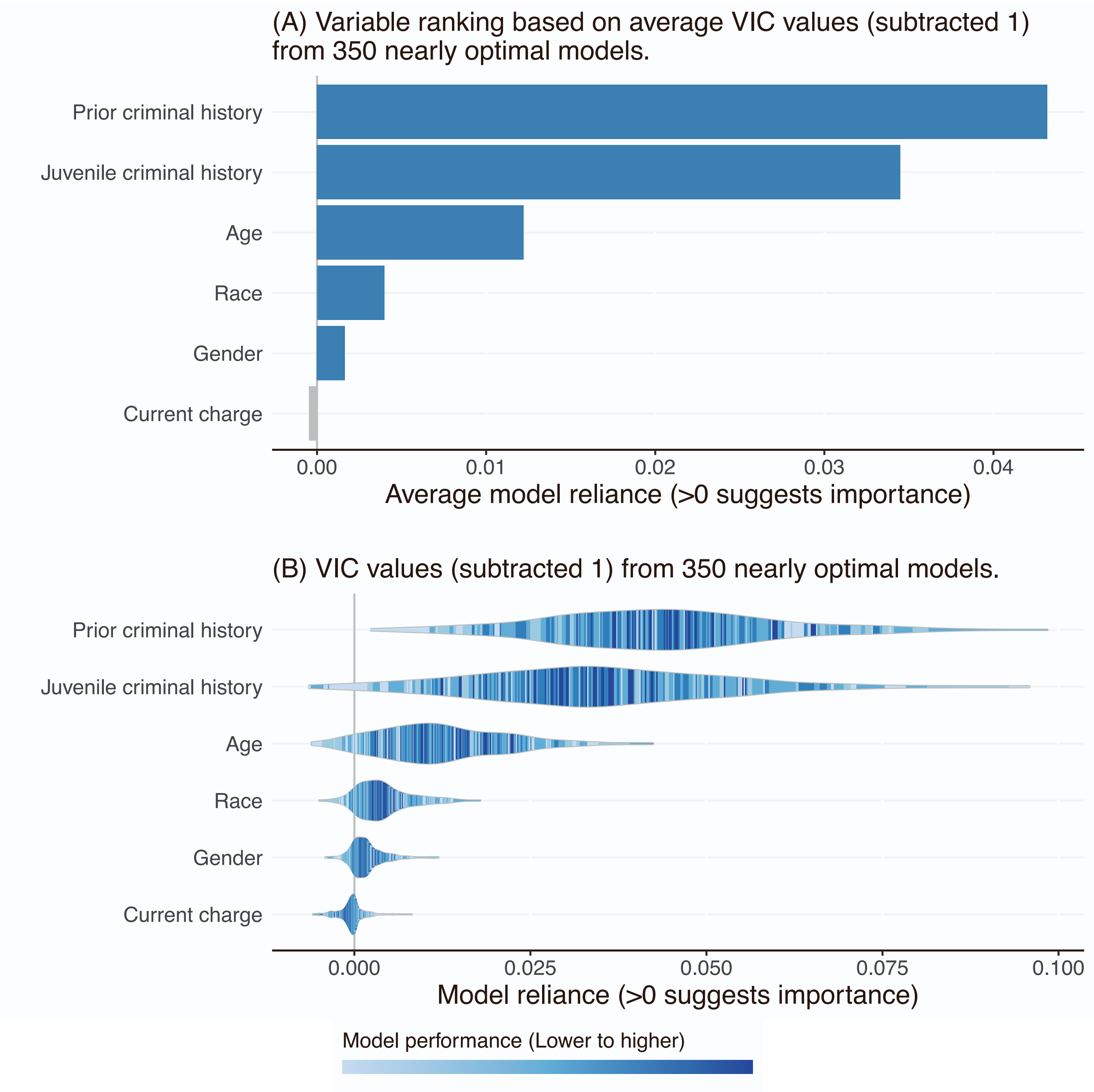

Figure S2. Spearman correlation among variables in the MIMIC study, arranged by variance inflation factor from the minimum-loss model. Absolute values larger than 0.4 are colored (in blue for positive correlation and red for negative correlation).

	Glucose	WBC	SpO2	Platelet	Temperature	Respiration rate	Age	Lactate	Potassium	Heart rate	BUN	Creatinine	SBP	DBP	Anion gap	MAP	Sodium	Bicarbonate	Chloride	Hematocrit
WBC	0.14																			
SpO2	0.01	0.03																		
Platelet	0.04	0.29	−0.05																	
Temperature	0.01	0.12	0.06	0																
Respiration rate	0.05	0.11	−0.25	0.09	0.12															
Age	0.07	0.01	−0.06	−0.05	−0.16	0.07														
Lactate	0.12	0.12	0.08	−0.11	0.02	0.01	−0.02													
Potassium	0.08	0.08	0.01	−0.03	−0.07	−0.05	0.08	0.05												
Heart rate	0.08	0.17	−0.05	0.03	0.27	0.29	−0.21	0.1	0											
BUN	0.14	0.03	−0.07	−0.08	−0.18	0.11	0.34	0.03	0.3	−0.07										
Creatinine	0.1	0.01	−0.06	−0.08	−0.11	0.08	0.17	0.07	0.29	−0.08	0.74									
SBP	0.08	−0.06	0.01	0.07	0.05	−0.03	0	−0.04	−0.08	−0.09	−0.03	0								
DBP	0.01	−0.07	−0.03	0.07	0.02	0.01	−0.36	0	−0.11	0.2	−0.2	−0.11	0.49							
Anion gap	0.16	0.11	−0.07	0.14	−0.04	0.16	−0.02	0.18	0.1	0.09	0.34	0.42	0.07	0.07						
MAP	0.06	−0.04	0.03	0.06	0.05	−0.03	−0.22	0.01	−0.11	0.09	−0.16	−0.1	0.76	0.85	0.07					
Sodium	−0.06	−0.04	0.01	0	0.03	0.02	0	0.01	−0.19	−0.05	0.01	0	0.11	0.08	0	0.1				
Bicarbonate	−0.06	−0.13	−0.12	0.09	−0.01	−0.07	0.04	−0.17	−0.11	−0.13	−0.13	−0.18	0.11	0.05	−0.42	0.06	0.11			
Chloride	−0.08	0.03	0.19	−0.19	0.06	−0.07	0	0.05	−0.01	0.01	−0.09	−0.1	−0.07	−0.06	−0.24	−0.04	0.54	−0.41		
Hematocrit	0.03	0.1	−0.15	0.13	0.01	0	−0.15	0.06	−0.04	−0.06	−0.2	−0.08	0.12	0.28	0.14	0.23	0.12	0.1	−0.11	
Hemoglobin	0.02	0.08	−0.13	0.09	0.03	−0.02	−0.17	0.06	−0.08	−0.07	−0.23	−0.11	0.13	0.29	0.12	0.25	0.1	0.08	−0.1	0.97

BUN: blood urea nitrogen; DBP: diastolic blood pressure; MAP: mean arterial pressure; SBP: systolic blood pressure; SpO_2: peripheral capillary oxygen saturation; WBC: white blood cell.

Figure S3. Visual summary of MIMIC study results from the VIC analysis. VIC values were subtracted 1 such that zero value or lower indicate unimportance. (A) Average values from the 350 nearly optimal models. Five of the top seven variables (i.e., except MAP and chloride) by average VIC values were also ranked top seven by average ShapleyVIC values. (B) Distribution of VIC values (subtracted 1) and the corresponding model performance.

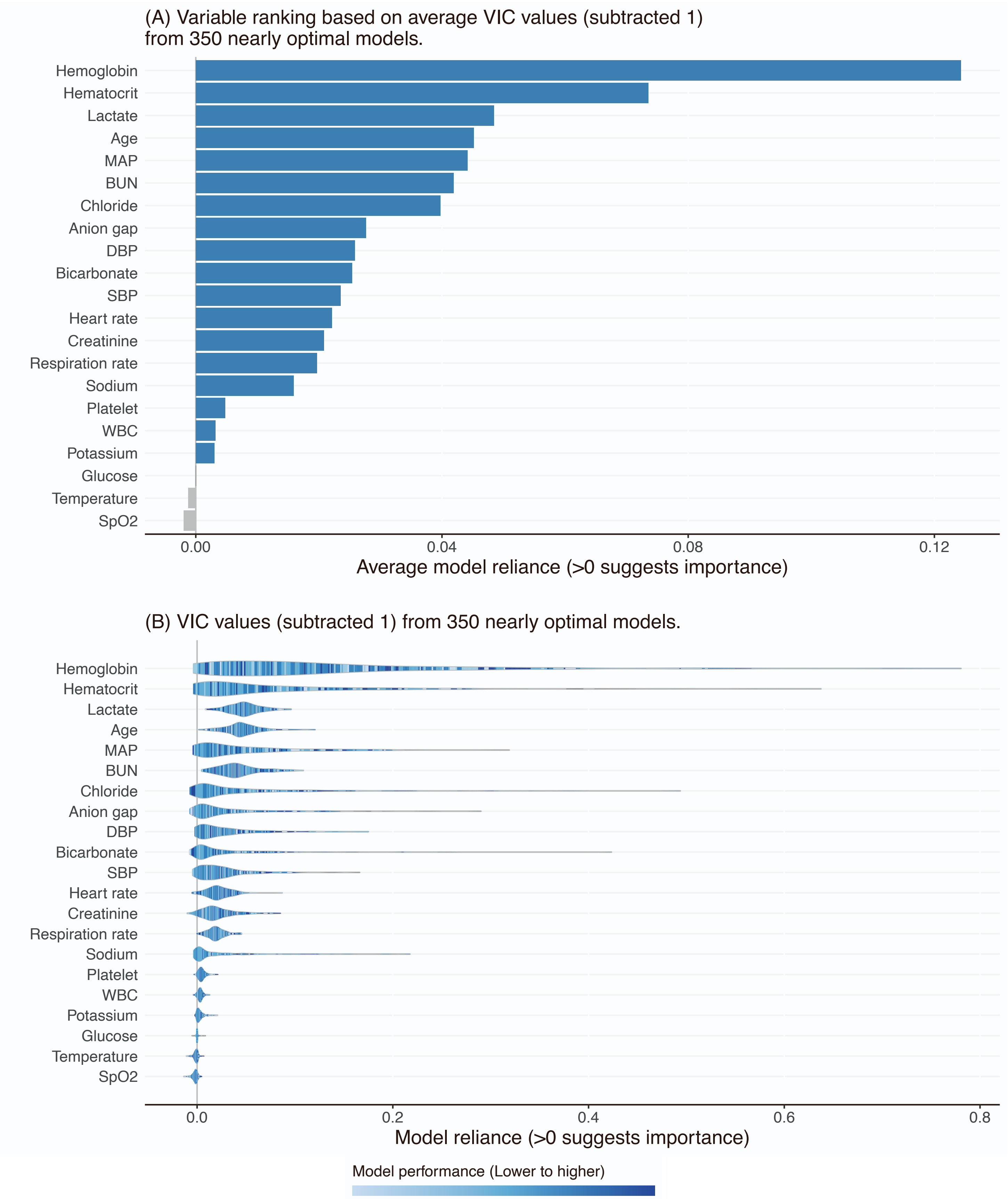

BUN: blood urea nitrogen; DBP: diastolic blood pressure; MAP: mean arterial pressure; SBP: systolic blood pressure; SpO_2: peripheral capillary oxygen saturation; WBC: white blood cell.
